# Comprehensive Physiotherapeutic Management of Cervical and Lumbar Disc Disease: A Case Study

**DOI:** 10.7759/cureus.52543

**Published:** 2024-01-19

**Authors:** Rutuja G Sawalkar, Vrushali Athawale, Tejaswini Fating

**Affiliations:** 1 Community Health Physiotherapy, Ravi Nair Physiotherapy College, Datta Meghe Institute of Higher Education and Research, Wardha, IND; 2 Oncology Physiotherapy, Ravi Nair Physiotherapy College, Datta Meghe Institute of Higher Education and Research, Wardha, IND

**Keywords:** degenerative spine disease, physical therapy rehabilitation, lumbar disc disease, cervical disc disease, intervertebral disc disease

## Abstract

This case report focuses on a 75-year-old male diagnosed with cervical and lumbar disc disease, common conditions associated with intervertebral disc degeneration. The study aims to highlight the significance of physiotherapy in managing these conditions. The patient presented with neck and lower back pain radiating to the limbs which was managed conservatively with analgesics and physiotherapy. The physiotherapeutic intervention included a tailored regimen involving cryotherapy, transcutaneous electrical nerve stimulation (TENS), strengthening exercises, task-specific training, and the use of a stabilometric platform. The pre- and post-intervention assessments revealed improvements in range of motion, muscle strength, and various outcome measures, emphasizing the effectiveness of the holistic physiotherapy approach. The case underscores the importance of physiotherapy in addressing degenerative disc diseases, offering insights into specific interventions such as cryotherapy, targeted exercises, and advanced technologies like stabilometric platforms. This study contributes to the existing literature on the role of physiotherapy in managing cervical and lumbar disc diseases, emphasizing the need for patient education and a comprehensive approach to improve overall physical functioning.

## Introduction

Degeneration of the intervertebral discs, which results in discomfort in the arms, legs, back, and neck, is a common ailment known as intervertebral disc disease. A person's experience of the deterioration and accompanying discomfort is anticipated to vary, affecting around 5% of the population in affluent nations annually [1]. Patients with this illness have some degree of spinal degeneration beyond the age of 40, with older adults being most affected. The age-related degeneration of intervertebral discs is caused by several factors, including senescence of the cells, loss of viable cells, and decreased nutrition [2]. The degenerative processes of the intervertebral disc are one of the main causes of morbidity and reduced nutrition [3]. The most common regions affected are the cervical and lumbar.

Pain and discomfort are caused by a degenerative disc in the neck. It can result in a loss of vertebral body height and undue strain on the failing disc, which can produce symptoms including tingling, numbness, and weakness in the hands and arms. This is usually caused by the disc material degenerating. If the sickness is asymptomatic, imaging tests like magnetic resonance imaging (MRI) and X-rays can be used to diagnose it [4]. Injuries to the spine, aging, genetic susceptibility, and lifestyle choices, including smoking and obesity, are among the causes of cervical disc disease. These elements play a role in the cervical discs' deterioration, which can cause pain and suffering [5,6]. A prevalent disorder affecting the intervertebral discs in the lower back is lumbar disc disease, commonly referred to as lumbar degenerative disc disease. A frequent ailment that causes lower back discomfort, leg pain, and weakness in lower limbs is lumbar disc disease, commonly referred to as lumbar degenerative disc disease. Inactivity, weakened back muscles, and hard lifting are risk factors. Numbness, limb weakness, and back discomfort are among the symptoms [7]. Given the age-related rise in spinal degenerative disease, lumbar disc disease is a common disorder with an estimated 27.3% prevalence [8].

The efficiency of physiotherapeutic techniques in the management of degenerative disc disease in the cervical and lumbar region, with a focus on the value of manual therapy and stabilometric platform exercises, is done [9,10]. The function of physical therapy in treating cervical and lumbar disc disease focuses on applying it to both the acute and chronic stages of the illness. This paper presents a case study involving a 75-year-old male patient diagnosed with cervical and lumbar disc disease. The primary objective of this study is to underscore the significance of physiotherapy in addressing this prevalent condition despite the limited number of conducted research studies on the subject.

## Case presentation

Patient’s information

A 75-year-old male, right-hand dominant, started experiencing pain in the back of the neck radiating bilaterally to the upper limbs in the past five months including pain over the lower back radiating bilaterally to the lower limbs. He visited a private practitioner, where he was prescribed analgesics and was referred to Acharya Vinoba Bhave Rural Hospital (AVBRH). With medications, his pain was reduced for the time being. Investigations like X-rays and MRI was done. In X-ray, cervical and lumbosacral spine senile degenerative changes and multiple osteophytes were seen at various levels. In MRI spine, due to senile degenerative changes, cervical and lumbar disc disease is diagnosed. The patient was managed conservatively through analgesics and physiotherapy. Physiotherapy rehabilitation commenced with the implementation of a tailored regimen created especially for the patient's requirements.

Clinical findings

Before the commencement of the examination, informed consent was obtained from the patient, and he was examined. On examination, he was conscious, cooperative, and well-oriented to person, place, and time. The patient was afebrile and hemodynamically stable. The patient was seen in a supine lying posture with the head elevated to 30°. He was mesomorphic, with a BMI of 21 kg/m^2^. On observation, in lateral view, a forwarded neck and protracted shoulders were seen. The pain was insidious in onset, followed by a history of heavy lifting, but has gradually progressed over the past five months. The pain was associated with a tingling sensation in the bilateral upper and lower limbs. The pain was relieved with medication. All cervical and lumbar range of motion and manual muscle testing are noted in Tables 1, 2, respectively.

**Table 1:** Pre-rehabilitation ROM ROM: Range of motion

Joints	Pre-rehabilitation ROM
Cervical	
Flexion	0°-20°
Extension	0°-25 °
Lateral flexion	0°- 10°
Lumbar	
Flexion	0°-30°
Extension	0°-10°
Lateral flexion	0°-15°

**Table 2:** Pre-rehabilitation MMT (MRC grading) MMT: Manual muscle testing; MRC: Medical Research Council Grade 0: no contraction; Grade 1: flicker of contraction; Grade 2: full range of motion actively in anti-gravity position; Grade 3: full range of motion actively against gravity; Grade 4: full range of motion actively against gravity with minimal resistance; Grade 5: full range of motion actively against gravity with maximal resistance

Joints	Pre-rehabilitation MMT
Cervical	
Flexion	3/5
Extension	3/5
Lateral flexion	3/5
Lumbar	
Flexion	3/5
Extension	3/5
Lateral flexion	3/5

Diagnostic assessment

**Figure 1:**
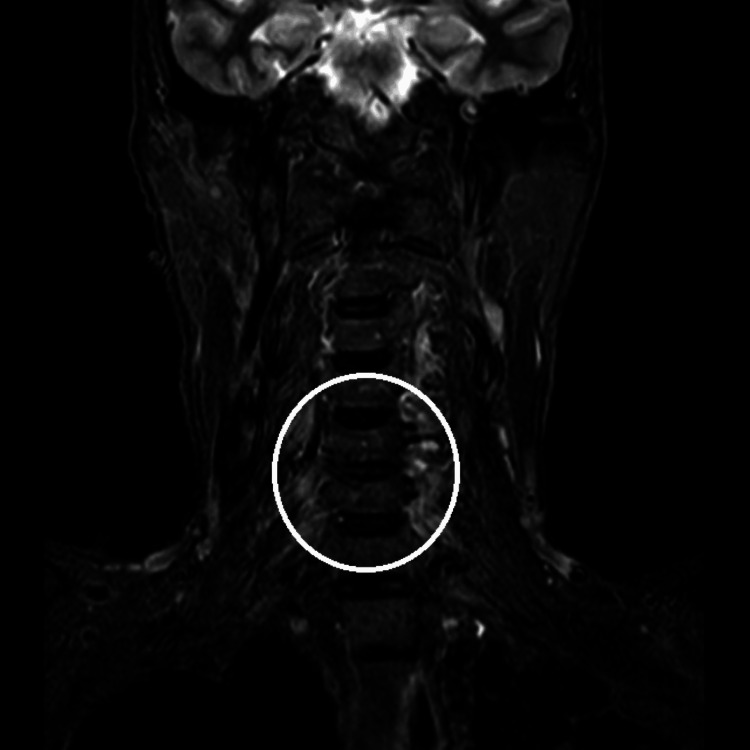
MRI cervical spine MRI: Magnetic resonance imaging The circle denotes C3-C4 diffuse disc bulge indenting over the anterior thecal sac, causing mild narrowing of the neural foramina indenting over the exiting nerve roots.

**Figure 2:**
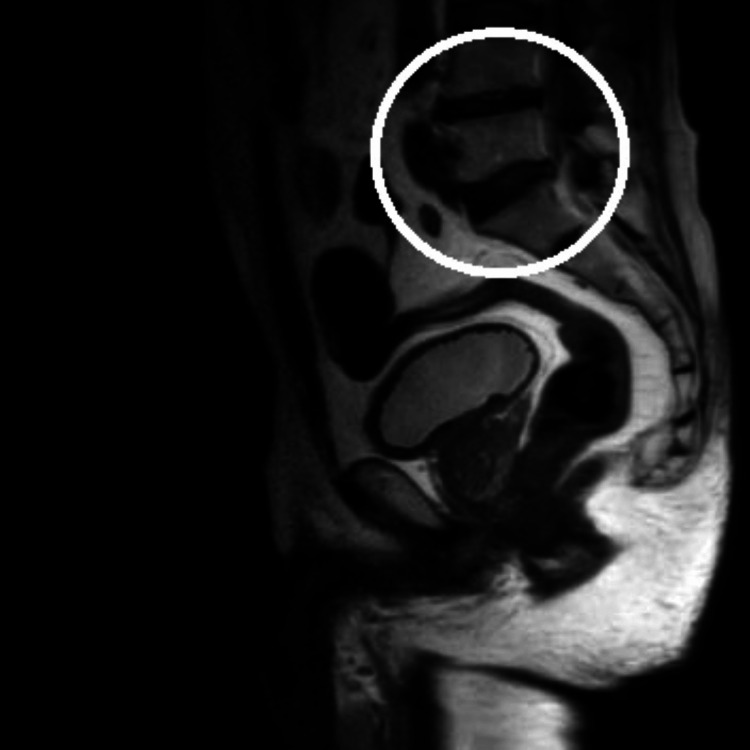
MRI lumbar spine MRI: Magnetic resonance imaging The circle denotes reduced disc height at the L4-L5 level and disc desiccation with diffuse disc bulges noted at the L4-L5 level.

Investigations like complete blood count, X-ray, and MRI were done. In X-ray, cervical and lumbosacral spine senile degenerative changes and multiple osteophytes were seen at various levels. In MRI, spinal C3-C4 diffuse disc bulge indenting over the anterior thecal sac causes mild narrowing of the neural foramina indenting over the exiting nerve roots (Figure 1). Reduced disc height at the L4-L5 level and disc desiccation with diffuse disc bulges were noted at the L4-L5 level (Figure 2).

Physiotherapy intervention

A physiotherapy rehabilitation protocol was planned, which is summarized in Table 3.

**Table 3:** Physiotherapy rehabilitation TENS: Transcutaneous electrical nerve stimulation; ADLS: activities of daily living; Hz: hertz

Problem Identified	Goal	Treatment Strategy	Intervention	Progression
Patient and family education	To augment and sustain a patient's favorable outlook toward their treatment regimen, facilitating an expedited recuperation process	The engagement of the therapist with both the patient and their family	The patient, accompanied by his family, received comprehensive explanations about the patient's condition and was informed about the critical role of physiotherapy intervention.	The home program was explained.
Pain	To relieve pain	Cryotherapy	Icing given for 10-20 min	-
	To reduce radiating pain in limbs	TENS	TENS given for 10 min with 60 Hz	
Weakness in muscles	To increase the strength of the muscles	Strengthening exercises	Static strengthening exercises for cervical and lumbar flexors, extensors, and lateral flexors	Strengthening exercises for cervical with blue theraband and for lumbar with weight cuffs of 1 kg.
			Engaging in strengthening routines using a yellow theraband.	
Difficulty in activities of daily living	To facilitate the ADLS	Task-specific training	Functional reach-outs with Swiss ball	
Difficulty in balance	To improve balance	Exercises on a stabilometric platform	Enhancing balance through training involves employing the visual feedback technique on a force platform during training sessions. In this mode, patients sway their bodies in various directions on the force platform, guided by visual feedback displayed on the screen in front of them. The screen features a ball situated on a platform with mazes. The ball's movement corresponds to the adjustments made by the patient on the force platform, mirroring the applied pressure.	-
Postural deviations	To improve posture	Posture correction and training	Mirror therapy	-

Follow-up and outcome measures

An organized physical therapy interventional protocol was started. For three weeks, a follow-up was carried out once per week. The findings of the outcome measure are shown in Tables 4-6.

**Table 4:** Post-rehabilitation ROM ROM: Range of motion

Joints	Post-rehabilitation ROM
Cervical	
Flexion	0°-40°
Extension	0°-30°
Lateral flexion	0°-40°
Lumbar	
Flexion	0°-50°
Extension	0°-15°
Lateral flexion	0°-15°

**Table 5:** Post-rehabilitation MMT (MRC grading) MMT: Manual muscle testing; MRC: Medical Research Council Grade 0: no contraction; Grade 1: flicker of contraction; Grade 2: full range of motion actively in anti-gravity position; Grade 3: full range of motion actively against gravity; Grade 4: full range of motion actively against gravity with minimal resistance; Grade 5: full range of motion actively against gravity with maximal resistance

Joints	Post-rehabilitation MMT
Cervical	
Flexion	4/5
Extension	4/5
Lateral flexion	4/5
Lumbar	
Flexion	4/5
Extension	4/5
Lateral flexion	4/5

**Table 6:** Pre-intervention and post-intervention outcome measures POMA: Performance-Oriented Mobility Assessment; Geriatric Pain Measure score: mild pain, <30; moderate pain, 30–69; severe pain, ≥70; Tinneti POMA tool score (risk of fall): ≤ 18, high; 19-23, moderate; ≥ 24, low

Outcome measures	Pre-intervention (day 1)	Post-intervention (day 21)
Geriatric Pain Measure	54	20
Tinetti POMA	20	26
Neck pain and disability scale	45	15

## Discussion

This case study presented a patient with lumbar and cervical disc disease. Reducing patient's difficulties in carrying out everyday tasks was the main goal. The patient's current issues were the focus of the physiotherapy treatment plan, which was also created with future challenges in mind. Cryotherapy is a potential treatment for pain in cervical and lumbar disc disease as it helps reduce inflammation and pain and improves function in patients with these conditions [11,12].

A targeted exercise regimen aimed at fortifying the deep neck flexors and scapulothoracic muscles, irrespective of their baseline strength levels. The recommended workouts focused on strengthening the endurance and functioning of important muscles of the cervical and lumbar region. Strengthening exercises of specialized deep neck flexors were included in the program [13]. The purpose of lumbar stabilization workouts is to improve the strength of the lumbar multifidi, internal obliques, and transversus abdominis, which are the intrinsic lumbar stabilizing muscles. Concurrently, lumbar dynamic strengthening exercises are done to activate the flexor muscles (rectus abdominis) and extensor muscles (erector spinae), therefore promoting a strategy for improving lumbar stability and strength [14,15]. The primary objective of task-specific training is to improve physical functioning, with a focus on enhancing neck muscle strength and endurance, improving sensorimotor function, and reducing discomfort due to pain [16].

Transcutaneous electrical nerve stimulation (TENS) is used for the treatment of pain. A continuous series of pulsed electrical currents is normally produced by TENS machines for pain relief, with frequencies ranging from 1 to 120 Hz and, in some cases, up to 200 Hz. This method works by sending regulated electrical impulses via the skin to reduce pain. Its goal is to modify and lessen the amount of pain that patients experience [17]. An equipment used in physical therapy to assist patients in improving their muscle strength, balance, and coordination is called a stabilometric platform. Using the platform, patients target specific muscle groups and enhance their overall function by engaging in a variety of activities under the supervision of a physiotherapist [18,19]. An essential part of treating cervical and lumbar disc degeneration is postural adjustment. For patients with cervical and lumbar disc degeneration, posture correction and training are important components of the treatment approach that enhance stability, lessen symptoms, and stop further damage [20].

## Conclusions

A 75-year-old man with lumbar and cervical disc disease was the subject of the case study. Increased range of motion and greater muscular strength were the two benefits of the physiotherapeutic intervention, which included the use of a stabilometric platform, focused workouts of specific muscle groups of the cervical and lumbar region, task-specific training, and cryotherapy. The goal of the approach was to improve total physical functioning in addition to treating the current symptoms. The study emphasizes the value of physiotherapy in the treatment of degenerative disc disorders and underscores the need for patient education.
